# Two novel Brugada syndrome-associated mutations increase K_V_4.3 membrane expression and function

**DOI:** 10.3892/ijmm.2015.2223

**Published:** 2015-05-26

**Authors:** TAO YOU, WEIKE MAO, BENZHI CAI, FAQIAN LI, HAODONG XU

**Affiliations:** 1 Department of Cardiology, The Second Affiliated Hospital of Soochow University, Suzhou, Jiangsu 215004, P.R. China; 2 Department of Pathology and Laboratory Medicine, University of Rochester Medical Center, Rochester, NY 14642, USA; 3 Aab Cardiovascular Research Institute, University of Rochester Medical Center, Rochester, NY 14642, USA; 4 Department of Pathology and Laboratory Medicine, David Geffen School of Medicine, UCLA, Los Angeles, CA 90095-1732, USA

**Keywords:** Brugada syndrome, K_V_4.3 mutants, K_V_4.3 functional expression

## Abstract

The human cardiac fast transient outward K^+^ channel is composed of the K_V_4.3 α subunit encoded by *KCND3* and the K^+^ channel-interacting protein 2 (KChIP2) β subunit, and determines the early repolarization of the action potential (AP). Two human mutations (G600R and L450F) in K_V_4.3 are associated with Brugada syndrome and they increase the K_V_4.3/KChIP2-encoded fast transient outward K^+^ current (I_to,f_) and cause the stable loss of the AP dome. However, the detailed mechanisms underlying the gain of I_to,f_ function by these two mutations are largely unknown. The experiments in the present study were undertaken to investigate the effect of these mutations and the underlying mechanism. Whole cell patch-clamp recording was performed in HEK-293 cells expressing K_V_4.3-wild-type (WT) and K_V_4.3 mutants with KChIP2. The two individual mutant-encoded currents were significantly increased but the kinetics of the channels affected by the two mutations were different. The two mutations slowed K_V_4.3/KChIP2-encoded channel inactivation; they did not increase the recovery from the K_V_4.3/KChIP2-encoded channel inactivation. Western blotting showed that total K_V_4.3 protein was significantly augmented in HEK-293 cells expressing the two individual mutants with KChIP2. Furthermore, immunofluorescence confocal microscopy demonstrated that the K_V_4.3 channel protein was expressed more in the cell membrane compared to the cytoplasm in cells that expressed individual mutants with KChIP2. Also, KChIP2 increased the amount of channel protein in the cell membrane of K_V_4.3 mutants significantly more than K_V_4.3-WT. Reverse transcription-polymerase chain reaction showed that K_V_4.3 mRNA was not significantly changed by individual mutations in the presence of KChIP2. Taken together, the present study revealed that the mutations cause a gain-of-function of K_V_4.3/KChIP2-encoded channels by increasing membrane protein expression and slowing channel inactivation.

## Introduction

Brugada syndrome (BrS) is a genetic heart disorder predominantly exhibiting an autosomal dominant pattern of inheritance and is thought to be responsible for 20% of sudden fatalities in people <50 years of age with apparently structurally normal hearts (1). It is characterized by a dynamic coved-type ST-segment elevation in the right precordial leads (V1–V3) of the electrocardiogram (ECG), and clinically by an increased risk of ventricular tachycardia (VT) or ventricular fibrillation (VF) and sudden fatality (2). The phenotype and penetrance of the disease appear to be associated with factors that alter the balance of outward and inward currents at the end of phase I of the epicardial ventricular action potential (3). In BrS, it is believed that the arrhythmic substrate is the result of increased heterogeneity of the currents involved in the phase I repolarization of the action potential (AP) in the right ventricle (4). Electrophysiological evidence indicates that the basis for arrhythmogenicity is the loss of the AP dome in certain epicardial sites (5). This leads to increased epicardial dispersion of repolarization, enabling local re-excitation via phase II reentry (6). Multiple mutations of genes, *SCN5A*, *SCN1B*, *SCN3B*, *GPD1L*, *CACNA1C*, *CACNB2*, *KCNE3* and *KCND3*, have been shown to decrease cardiac Na^+^ and Ca^2+^ channel activity or increase fast transient outward K^+^ channel activity, and they are linked to BrS (7).

The voltage-gated cardiac fast transient outward K^+^ current (I_to,f_) plays a predominant role in determining the initial repolarization of AP. It is well-documented that the human cardiac I_to,f_ channel is composed of the α subunit/K_V_4.3 and β subunit/K^+^ channel-interacting protein 2 (8). The expression of the K_V_4.3 channel in normal or failing human and canine hearts exhibits a transmural gradient in the left ventricle (9), which is responsible for the transmural electrophysiological heterogeneity (10). Accordingly, an early repolarization abnormality as a result of decreased expression and dysfunction of the K_V_4.3 channel in a variety of heart diseases, such as myocardial infarction and heart failure, contributes to the pathogenesis of life-threatening cardiac arrhythmias (11). Recently, two missense mutations have been identified in the K_V_4.3-encoded gene, *KCND3*, from patients with BrS. The two individual mutants have been shown to produce a gain of I_to,f_ function in HEK-293 cells when co-expressing with KChIP2 (12). Simulations using a Luo-Rudy II action potential mode demonstrated the stable loss of the AP dome as a result of the increase of I_to,f_ maximal conductance associated with heterozygous expression of either the L450F or G600R mutant of K_V_4.3 in the presence of KChIP2 (12). However, the individual role of the mutants and KChIP2 in the gain-of-function of the K_V_4.3 channel is unclear. The present study demonstrated that two mutations are sufficient to increase the K_V_4.3 current by increasing membrane expression, but KChIP2 also participates with the mutation-related BrS by increasing K_V_4.3 protein expression and regulating channel kinetics.

## Material and methods

### Plasmids and reagents

**Figure 1:**
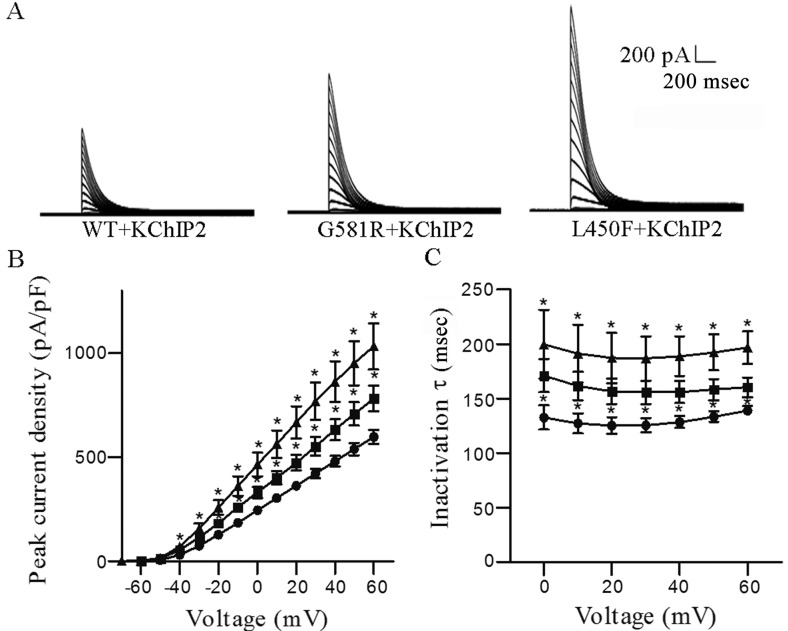
Effects of G581R and L450F on K_V_4.3/KChIP2-encoded K^+^ currents. (A) In HEK-293 cells transfected with plasmids encoding K_V_4.3-WT, K_V_4.3-G581R or K_V_4.3-L450F together with KChIP2, K_V_4.3-G581R and K_V_4.3-L450F significantly increased the transient outward K^+^ current density compared with K_V_4.3-WT. (B) The current-voltage association for the transient outward K^+^ currents in cells expressing K_V_4.3-WT (n=12), K_V_4.3-G581R (n=11) or K_V_4.3-L450F (n=12) with KChIP2. Two mutations significantly increase transient outward K^+^ current densities in HEK-293 cells expressing K_V_4.3-G581 and K_V_4.3-L450F in the presence of KChIP2 at voltages from −40 to +60 mV. (C) Two mutations significantly slow the K_V_4.3-G581R/KChIP2-encoded (n=11) or K_V_4.3-L450F/KChIP2-encoded (n=7) channel inactivation at voltages from 0 to +60 mV compared to K_V_4.3-WT (n=12). •, K_V_4.3-WT/KChIP2; ■, K_V_4.3-G581R/KChIP2; ▲, K_V_4.3-L450F/KChIP2. (C–E) ^*^P<0.05 vs. K_V_4.3-WT/KChIP2. The error bars represent standard error of the mean for the indicated number of cells from each group. WT, wild-type.

cDNA plasmids-encoding K_V_4.3 and KChIP2, respectively, were kindly provided by Dr Jeanne M. Nerbonne at Washington University School of Medicine in St. Louis (MO, USA). Alignment by NCBI Blast software (http://blast.ncbi.nlm.nih.gov/Blast.cgi) showed that >90% of the amino acids of rat (NCBI protein, NP_001257891.1, 10-Aug-2014) and human (NCBI protein, NP_004971.2, 25-May-2014) K_V_4.3 are identical (Fig. 1B). Two targeted mutations in rat *KCND3* were generated using the QuickChange II XL site-directed mutagenesis kit (Agilent, Santa Clara, CA, USA) according to the manufacturer’s instructions. Rat *KCND3*, with two individual mutations corresponding to BrS in humans, was confirmed by sequencing of the constructs using the Big T3 terminator kit (Applied Biosystems, Foster City, CA, USA) (Fig. 1A) and they encode K_V_4.3-G581R and K_V_4.3-L450F, respectively. The following primers were used for polymerase chain reaction (PCR): rK_V_4.3-G581R, 5′AAAGCAGACGATCGACTGAGACCAA-3′ [nucleotides (nt) 1821–1855]; and rK_V_4.3-L450F, 5′GCGCAATGGACT CTTCAATGAAGCTCTGG-3′ (nt 1427–1455). The underlined letters (TC and T) refer to the base pair changes corresponding to the rat K_V_4.3 mutation G580R and L450F, respectively.

### Cell culture and transfection

HEK-293 cells were cultured in Dulbecco’s modified Eagle’s medium supplemented with 10% fetal bovine serum (Sigma-Aldrich, St. Louis, MO, USA), 100 U/ml penicillin and 100 *µ*g/ml streptomycin (Life Technologies, Grand Island, NY, USA). The cells were maintained at 37°C in a 5% CO_2_ incubator, and passaged every 3 days at confluence. Prior to transfection, cells were seeded in 6-well plates at a density of 5×10^5^ cells/ml. After 24 h, a total of 1.0 *µ*g plasmid DNA was diluted in transfection medium using Lipofectamine 2000 reagent (Invitrogen, Grand Island, NY, USA) according to the manufacturer’s instructions.

### Western blot analysis

HEK-293 cells were lysed on ice with ice-cold Lysis buffer (Cell Signaling Technology, Boston, MA, USA) following rinsing with phosphate-buffered saline (PBS). Cell lysates were subsequently collected and prepared by passing through a 25-G needle connected to a 1-ml syringe 10 times before centrifugation at 17,000 × g for 10 min at 4°C. Supernatants were harvested and the protein concentration was determined using the Bradford method and a spectrophotometer. Equal amounts of protein were loaded onto a polyacrymide gel and run at 80–120 V for 2 h, and were subsequently transferred onto a polyvinyl fluoride membrane overnight. The membranes were washed in PBS three times for 5 min each, and blocked with 5% skimmed powdered milk for 1 h. Subsequently the membranes were incubated with a monoclonal antibody against K_V_4.3 (mouse anti-rat, 1:1,000; Cat. no. 75-017), KChIP2b (mouse anti-rat, 1:2,000; Cat. no. 75-004) (from NeuroMab, Davis, CA, USA) or glyceraldehyde-3-phosphate dehydrogenase (GAPDH) (mouse anti-rabbit, 1:4,000; Cat. no. CB1001; Millipore, Billerica, MA, USA) at 4°C overnight and washed 3 times prior to incubation with horseradish peroxidase (HRP)-conjugated goat anti-mouse secondary antibodies (polyclonal, 1:3,000; Cat. no. AP181P; Millipore) for 1 h at room temperature. ECL chemiluminescent reagent (Perkin Elmer, Waltham, MA, USA) was the chemiluminescent substrate used for chemiluminescence-based immunodetection of HRP.

### Reverse transcription (RT)-PCR

Total RNA was extracted from HEK-293 cells using the RNeasy Miniprep Plus kit (Qiagen, Valencia, CA, USA) according to the manufacturer’s instructions after transfection for 24 h with plasmids encoding K_V_4.3-wild-type (WT), or individual mutants with plasmids encoding KChIP2. RNA concentration was determined using a Bradford spectrometer at OD 260. Total RNA was stored in −80°C for further experiments. Reverse transcription was carried out with the High Capacity Reverse Transcription kit (Applied Biosystems) according to the manufacturer’s instructions. Subsequent PCR amplification was performed using the Bio-Rad MyCycler Thermal Cycler (Bio-Rad, Hercules, CA, USA). The following primers were used for amplification and detection of K_V_4.3: Forward, 5′-TTTGTCACACTCCGGGTCTTCCGT-3′, and reverse, 5′-TCATTGAGGAGCCCATTGCGCTTG-3′. *GAPDH* was determined using the following primer pairs: Forward, 5′-ACGGATTTGGTCGTATTGGG-3′, and reverse, 5′-CGCTCCTGGAAGATGGTGAT-3′. The PCR cycling conditions were: 94°C for 3 min; 27 cycles of 94°C for 30 sec, 55°C for 30 sec and 72°C for 30 sec; and 1 cycle of additional extension at 72°C for 7 min; and subsequently held at 4°C. The final concentration of all the reagents were: 1X Taq buffer, 0.2 mM of each dNTP, 0.2 *µ*M of each primer and 2 units of Taq polymerase. The expression of K_V_4.3 mRNA was normalized to the *GAPDH* level. The density of the K_V_4.3 mRNA and protein bands between groups was quantified using ImageJ (NIH, Bethesda, MA, USA).

### Whole-cell K_V_4.3 recording

**Figure 2:**
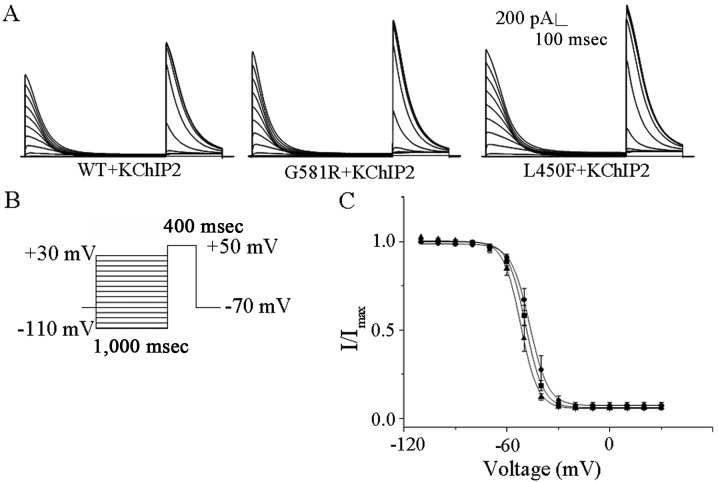
Effects of G581R and L450F on the steady-state inactivation kinetics of K_V_4.3/KChIP2-encoded K^+^ currents. (A) Currents showing steady-state inactivation curves recorded from K_V_4.3-WT, K_V_4.3-G581R and K_V_4.3-L450F with KChIP2. (B) The stimulation protocol used for steady-state inactivation. (C) Mean ± standard error (SE) normalized current amplitudes were plotted as a function of conditioning potential for the •, K_V_4.3-WT/KCHIP2; ■, K_V_4.3-G581R/KChIP2; and ▲, K_V_4.3F450F/KChIP2 encoded K^+^ currents. The solid lines represent the best (single) Boltzmann fits to the mean ± SE normalized data. G581R and L450F have no effects on the K_V_4.3/KChIP2-encoded channel voltage dependence of steady-state inactivation. The error bars represent standard error of the mean for ≥7 cells from each group.

**Figure 3:**
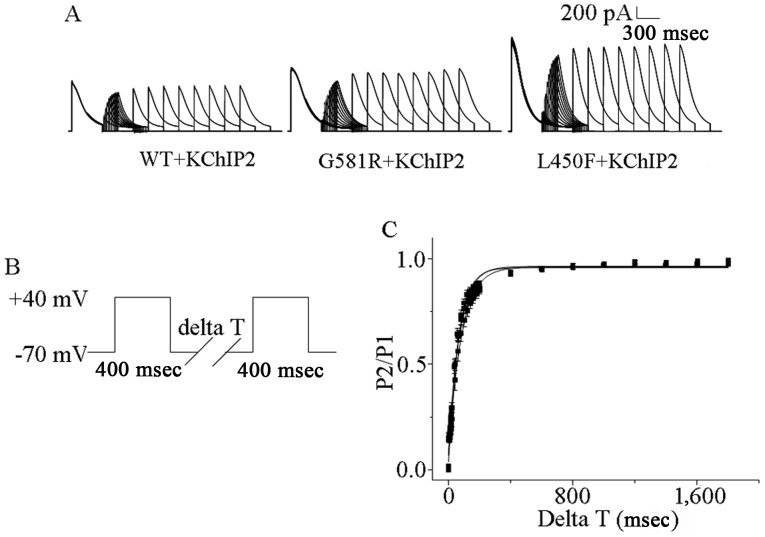
Effects of G581R and L450F on recovery from inactivation in K_V_4.3 with KChIP2. (A) Representative currents of recovery from inactivation recorded from G581R and L450F with KChIP2. (B) The stimulation protocol used for recovery from inactivation. (C) G581R and L450F do not promote the recovery of K_V_4.3/KChIP2 channel from inactivation compared with WT. The error bars represent standard error of the mean for ≥6 cells from each group.

Outward K^+^ currents in the HEK-293 cells were recorded in a voltage-clamp mode at room temperature (24°C). Experiments were conducted using a Axopatch 200B amplifier attached to a Dell desktop computer equipped with a DigiData 1322 series analog/digital interface and pClamp 10.0 software (all from Axon, Sunnyvale, CA, USA). Electrodes were pulled using a PC-10 vertical pipette puller (Narishige, East Meadow, NY, USA) and had a pipette resistance between 1.5 and 3.0 MΩ subsequent to filling with a recording pipette solution containing: 135 mM KCl, 1 mM MgCl_2_, 10 mM HEPES and 5 mM glucose (pH 7.2). The bath solution for the recording contained: 136 mM NaCl, 4 mM KCl, 1 mM CaCl_2_, 2 mM MgCl_2_, 10 mM HEPES and 10 mM glucose (pH 7.4). Only the data acquired from cells with an input resistance >0.7 GΩ were analyzed. Current densities were obtained from peak amplitudes normalized to cell capacitances. The voltage-dependent inactivation and recovery from inactivation were measured using the protocols shown in the Figs. 2 and 3. The voltage dependence of steady-state inactivation of the K_V_4.3-WT, K_V_4.3-G581R and K_V_4.3-L450F-encoded K^+^ currents in the presence of KChIP2 evoked from each conditioning potential were measured and normalized to the current evoked from −70 mV (in the same cell). Each sweep was applied with 10 sec intervals. Data were obtained at different sampling frequencies and the current signals were filtered simultaneously at 5 kHz prior to digitization and storage.

### Examination under immunofluorescence confocal micros- copy

HEK-293 cells were plated in 35-mm dishes overnight before transfection with plasmids containing cDNAs. Twenty-four hours after transfection, cells were fixed using 4% paraformaldehyde, washed 3 times with PBS and permeablized with 0.1% Triton X-100 (Sigma-Aldrich). After being blocked for 1 h with 10% normal goat serum (Invitrogen), the cells were washed and incubated with mouse anti-K_V_4.3 monoclonal antibody (1:200; NeuroMab) overnight. Four more wash steps of 5 min each were applied prior to incubation with Alexa Fluor 546 goat anti-mouse IgG (polyclonal, 1:2,000; Life Technologies) for 1 h at room temperature. Cells were subsequently washed and incubated in 300 nM 4′,6-diamidino-2-phenylindole (Sigma-Aldrich) for 5 min. The cells were washed 3 times. Subsequently, the cells were examined and images were captured using an IX-81 laser confocal microscopy (Olympus, Tokyo, Japan). Images were captured at magnification, x400 and analyzed using NIH ImageJ software. Cell membrane localization of WT and mutant K_V_4.3 was determined by calculating the ratio of membrane to cytoplasmic fluorescence intensity as follows: Membrane/cytosolic ratio = (peak membrane intensity - background)/(mean cytosolic intensity - background). The intensities were determined by a line scan through each cell. The cytosolic intensity was calculated as the mean intensity over at least two membrane thicknesses inside the cell. The background was determined as the mean intensity at two membrane thicknesses away from the cell. The membrane and cytosolic intensity were measured per cell in a blinded fashion, and the mean ratio for each mutant was normalized by the mean WT ratio for the same day.

### Statistical analysis

All the values are presented as the means ± standard error of the mean. Two-tailed Student’s t-test or one-way analysis of variance (multiple groups) followed by the Dunnett’s test (for single comparisons) was used to compare the difference between various groups. P<0.05 was considered to indicate a statistically significant difference.

## Results

### K_V_4.3-G581R and K_V_4.3-L450F cause a gain-of-function of transient outward K^+^ currents

In order to determine the effects of individual mutations on K_V_4.3/KChIP2-encoded K^+^ channels, HEK-293 cells were transfected with plasmids encoding K_V_4.3-WT, K_V_4.3-G581R and K_V_4.3-L450F with KChIP2 and green fluorescent protein (GFP). GFP expression allowed visualization of cells for whole-cell recording. Voltage-gated transient outward K^+^ currents were evoked by a 4,500 msec depolarizing pulse between −70 and +60 mV in 10 mV increments from a holding potential of −70 mV. The transient outward K^+^ current was significantly increased in HEK-293 cells expressing K_V_4.3-G581R or K_V_4.3-L450F with KChIP2 (Fig. 1A). In comparison with K_V_4.3-WT plus KChIP2, K_V_4.3-G581R and K_V_4.3-L450F plus KChIP2-encoded peak current densities were significantly (P<0.05) increased from −30 to +60 mV (Fig. 1B). For example, they were increased by 33.7 and 79.4%; from 480.7±26.1 pA/pF (n=12) to 642.9±54.1 pA/pF (n=11) and 862.6±98.5 pA/pF (n=12), respectively, at +40 mV (Fig. 1B).

### K_V_4.3-G581R and K_V_4.3-L450F influences the kinetics of transient outward K^+^ currents

Analysis of the decay phases of the outward K^+^ currents-encoded by K_V_4.3-WT, K_V_4.3-G581R and K_V_4.3-L450F with KChIP2 revealed that current decay is well-described by the sum of one exponential and neither time constant exhibits any appreciable voltage dependence (Fig. 1C). Each individual mutation significantly (P<0.05) slowed K_V_4.3-L450F + KChIP2 or K_V_4.3-G581R + KChIP2-encoded channel inactivation from 0 to +60 mV compared to K_V_4.3-WT (Fig. 1C), confirmed by mean ± standard error (SE) time constants of inactivation (τ_inactivation_) at 188.8±18.3 msec (L450F) and 155.9±10.0 msec (G581R) compared to 128.5±5.6 msec (WT).

The voltage dependences of steady state inactivation of K_V_4.3-WT, K_V_4.3-G581R and K_V_4.3-L450F with KChIP2 were examined during 400 msec depolarization to +50 mV after 1 sec conditioning prepulses to potentials between −110 and +30 mV; the protocol (Fig. 2B) is shown below the current records in Fig. 2A. The steady state inactivation data for transient outward currents were well-described by a single Boltzmann equation. The individual mutations did not affect the values of V_1/2_ and K (data not shown), indicating that they did not significantly affect the steady-state voltage-dependent inactivation of K_V_4.3/KChIP2-encoded channels (Fig. 2C).

To examine the effects of two individual mutations on the time dependency of recovery from steady state inactivation of K_V_4.3 alone and with KChIP2-encoded channels, HEK-293 cells expressing K_V_4.3-WT, K_V_4.3-G581R or K_V_4.3-L450F with KChIP2 were first depolarized to +40 mV for 400 msec to inactivate the currents, subsequently hyperpolarized to −70 mV for varying times ranging from 2 to 7,500 msec, and finally stepped to +40 mV to activate the currents and assess the extent of recovery; the protocol (Fig. 3B) is illustrated below the K^+^ currents (Fig. 3A). Analysis of the normalized current amplitudes as a function of the recovery time (interpulse interval) revealed that the time courses of recovery of K_V_4.3-WT, K_V_4.3-G581R or K_V_4.3-L450F with KChIP2-encoded K^+^ currents at −70 mV are well-described by single exponentials. The mean ± SE time constants of recovery (τ_recovery_) of K_V_4.3-WT, K_V_4.3-G581R or K_V_4.3-L450F with KChIP2-encoded K^+^ currents are 64.0±5.8 msec (n=6), 84.0±9.0 msec (n=7) and 71.2±5.9 msec, respectively, and there were no statistical differences among them.

### K_V_4.3-G581R and K_V_4.3-L450F increases the expression of K_V_4.3 protein

**Figure 4:**
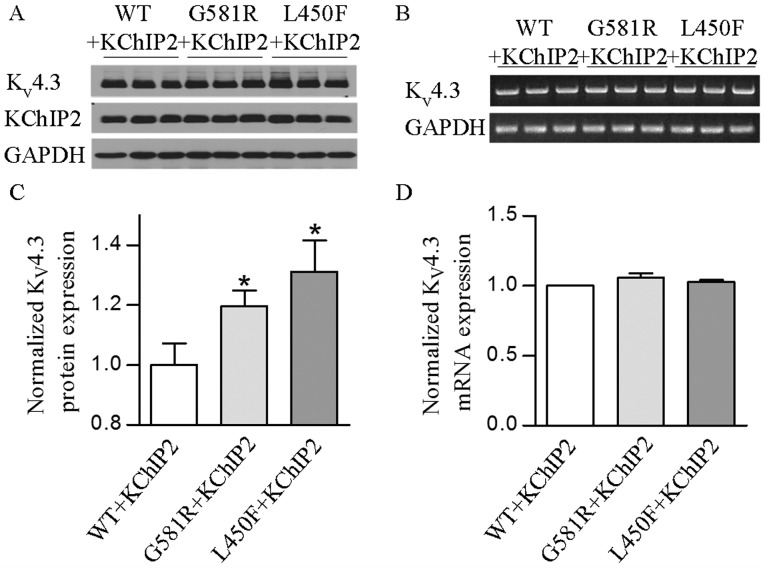
Effects of G581R and L450F on total K_V_4.3 channel expression in the presence of KChIP2. (A and C) Western blotting showed that total protein level of K_V_4.3 channel was significantly (^*^P<0.05) increased in HEK293 cells expressing K_V_4.3-G581R or K_V_4.3-L450F with KChIP2, compared with K_V_4.3-WT. The error bars indicate standard error of the mean (SEM) for ≥7 tests for each group. (B and D) Semi-quantitative revesre transcription-polymerase chain reaction detected no significant changes in K_V_4.3 mRNA levels among the HEK293 cells co-expressing K_V_4.3-WT, K_V_4.3-G581R and K_V_4.3-L450F with KChIP2. The error bars indicate SEM for ≥3 tests for each group.

The mutations slow the K_V_4.3/KChIP2-encoded channel inactivation, which contributes to the increase of a gain of channel function, but their influences on channel protein expression were not determined. Western blotting was performed on protein extracts from HEK-293 cells for analysis of K_V_4.3-WT, K_V_4.3-G581R and K_V_4.3-L450F with KChIP2 expression. The results showed that two individual mutations significantly (P<0.05 or P<0.01) increased the K_V_4.3 channel protein in the presence of KChIP2 (Fig. 4A and C). These findings indicate that increases of K_V_4.3 protein expression by two individual mutations also play an important role in the gain of K_V_4.3- and K_V_4.3/KChIP2-encoded K^+^ channels.

### K_V_4.3-G581R and K_V_4.3-L450F affects the localization of the K_V_4.3 protein

**Figure 5:**
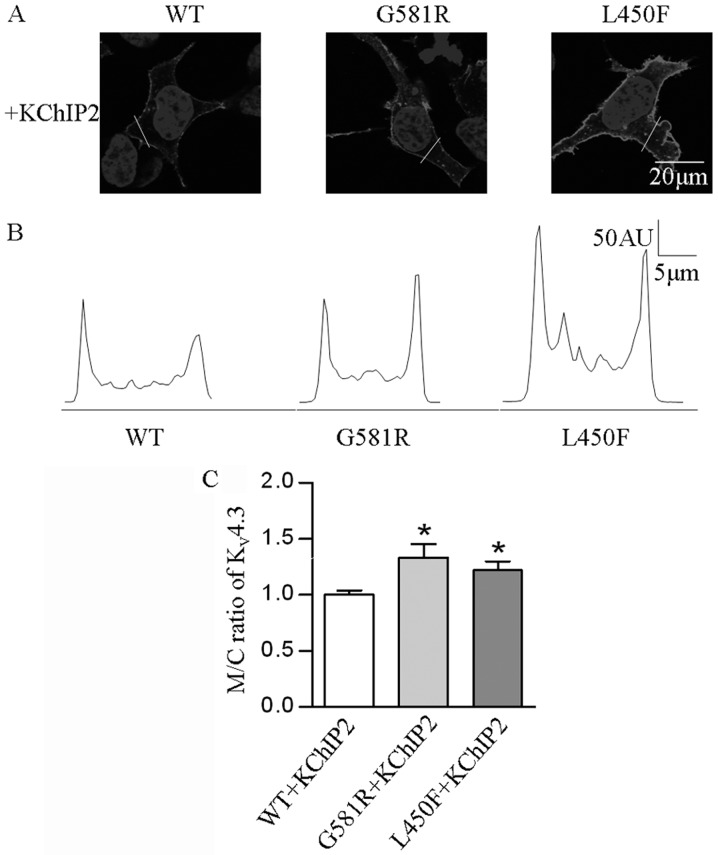
Effects of G581R and L450F on membrane localization of K_V_4.3 channel protein. (A) Representative confocal images of HEK-293 cells transfected with K_V_4.3-WT, K_V_4.3-G581R and K_V_4.3-L450F in the presence of KChIP2. (B) An intensity profile from a scan along the indicated line is shown below each image. (C) In the presence of KChIP2, confocal image analysis showed that G581R (n=10) and L450F (n=15) significantly (^*^P<0.05) increased the cell membrane to cytoplasmic intensity ratios of K_V_4.3 channel protein, compared with WT (n=13) ratio. The error bars represent standard error of the mean for the indicated number of cells from each group. AU, artbitrary units.

In order to determine if the two mutations affect the localization of the K_V_4.3 channel protein in HEK-293 cells expressing K_V_4.3-WT, K_V_4.3-G581R and K_V_4.3-L450F with KChIP2, confocal immunofluorescence microscopy was employed. The results revealed a significantly elevated (P<0.05) M/C ratio of K_V_4.3 channel in cells expressing the individual mutants and KChIP2, as compared with K_V_4.3-WT and KChIP2 (Fig. 5).

### K_V_4.3-G581R and K_V_4.3-L450F have no impact on K_V_4.3 mRNA levels

Whether the mutation-induced increase of total and cell surface K_V_4.3 protein occurred at its mRNA level and/or post-translational trafficking remains unclear. Thus, this was further investigated to determine if K_V_4.3-G581R and K_V_4.3-L450F can affect K_V_4.3 channel expression at the transcriptional level. Semi-quantitative PCR revealed that there was no significant change of the K_V_4.3 mRNA level in cells expressing each mutant plus KChIP2 when compared with K_V_4.3-WT plus KChIP2 (Fig. 4B and D). These findings indicate that the two mutations and KChIP2 do not play a regulatory role in the mRNA processing of the K_V_4.3 channel.

## Discussion

Taken together, our studies revealed that two BrS-associated *KCND3* mutations cause an increase of I_to,f_ currents by altering channel kinetics and increasing membrane protein expression. In comparison with previous studies (1), the effects of the mutations on the membrane protein expression were further studied. The impacts of the rat mutations on the K_V_4.3/KChIP2-encoded channel kinetics are slightly different from those in humans. Regardless, increased transient outward K^+^ channel functions by individual mutations are the same. The present findings provide more insights into the mechanisms of two *KCND3* mutations leading to BrS.

BrS is a genetic heart disease that is characterized by a specific ECG pattern and an increased risk of sudden cardiac fatality (13). The typical BrS electrocardiography findings present ST-segment elevation in leads V1 to V3 and right bundle branch block. Na^+^-channel blockers, such as ajmaline and flecainide (4,14), have been shown to reveal certain concealed EGC patterns in patients (7,15). Loss of the AP dome in the right ventricular epicardium is thought to be caused by ST-segment elevation in BrS. Abnormalities in electrical heterogeneity of the right ventricular epicardium is associated with the development of closely coupled premature ventricular contractions via a phase 2 reentrant mechanism that precipitates VT/VF (16).

The electrophysiological properties of ion channels are determined by multiple factors, such as an interactive subunit (8), temperature (17), pH value, ischemia, hypoxia, chemicals and cytokines (18,19). The preponderance of evidence shows that the genetic loss or gain-of-function of cardiac ion channels due to gene mutations may disrupt normal electrical activity and trigger cardiac arrhythmias, and even sudden cardiac fatality (1–3). Thus far, 9 BrS-associated gene mutations have been identified and include *SCN5A*, *GPD1L*, *CACNA1C*, *CACNB2*, *SCN1B*, *KCNE3*, *SCN3B*, *HCN4* and *KCND3* (1). Mutations in *KCNE3* were shown to induce the gain-of-function of the K_V_4.3 channel, which can underlie the development of BrS (20). Recently, Giudicessi *et al* (12) established a link between *KCND3* gene mutations and BrS, and demonstrated that two BrS-associated mutations (K_V_4.3-G600R and K_V_4.3-L450F) lead to the gain-of-function of I_to_, characterized by increasing I_to_ current density and the loss of the AP dome, which was suggested as a pathogenic substrate for BrS. However, the influences of the individual mutations on the expression and function of the K_V_4.3 channel, and the contribution of KChIP2 to the mutation-induced gain-of-function of the K_V_4.3 channel are unclear. Thus, whether the *de novo KCND3* mutations are sufficient to affect the expression and function of the K_V_4.3 channel were investigated. Our studies revealed that either of these two BrS-associated mutations in the *KCND3* gene produce an increase of transient outward K^+^ current by increasing K_V_4.3 protein expression, particularly in the cell surface. Additionally, co-expression with KChIP2 will promote an increase of peak current density, alter the channel kinetics and facilitate augmented protein expression of K_V_4.3 mutants. In addition, there were no significant changes in the mRNA level of the K_V_4.3 channel in the cells expressing individual mutants in conjunction with KChIP2. This suggests that the increased expression of the K_V_4.3 channel protein by the mutations may occur at the post-translational level as a result of increased protein stability or decreased protein degradation.

In conclusion, two novel BrS-related mutations in the *KCND3* gene were found to cause a gain-of-function of I_to_ by altering K_V_4.3 channel kinetics and membrane protein expression. These findings provide a comprehensive insight into the mechanism underlying BrS in two mutation-associated types. Further investigations are required to elucidate how the mutations enable K_V_4.3 to become more stabilized or what other post-translational modifications are involved in the increased membrane expression of the K_V_4.3 channel.
